# Editorial: Integrated omics approaches in the understanding of host-pathogen interactions

**DOI:** 10.3389/fcimb.2023.1215104

**Published:** 2023-05-25

**Authors:** Anis Rageh Al-Maleki, Kamil Braima, Naim Asyraf Rosli

**Affiliations:** ^1^ Medical Microbiology Department, Faculty of Medicine, Universiti Malaya, Kuala Lumpur, Malaysia; ^2^ Department of Medical Microbiology, Faculty of Medicine and Health Sciences, Sana’a University, Sana’a, Yemen; ^3^ Global and Tropical Health Division, Menzies School of Health Research and Charles Darwin University, Darwin, NT, Australia

**Keywords:** omics, host-pathogen interactions, genomics, transcriptomics, proteomics, metabolomics, metabolic pathways, microbial infection

Technologies with high throughput have fundamentally altered medical research (Khan et al., 2019). In recent years, the concept of “omics” has emerged, which refers to the study of various biological molecules such as DNA, RNA, proteins, and metabolites (Subramanian et al., 2020). The area of “integrated omics” was created as a result of the development of many methods, enabling for comprehensive genome-wide association investigations and techniques to better understand the complexities of human diseases (Wörheide et al., 2021). In recent years, biological researchers have frequently used omics technologies, such as proteomics, transcriptomics, and metabolomics, in their regular research processes to study host-pathogen interaction (Jean Beltran et al., 2017; Escudero-Perez et al., 2023). The interaction can be beneficial, detrimental, or neutral for either of the organisms involved (Gupta et al., 2017). Understanding the mechanisms involved in host-pathogen interaction is essential in developing effective treatment strategies and critical in preventing the spread of infections, especially in hospitals and other healthcare settings (Chai et al., 2020; Klein and Hultgren, 2020). We present a summary of intriguing papers that pertain to this research area.

An article on this Research Topic has extensively discussed the importance of integrated omics approaches in decoding virulence and fitness of Gram-negative bacteria, which is the possible outcome of host-pathogen interaction. An integrated omics technology, which enables researchers to identify the crucial genes, proteins, and metabolites involved in the network of interactions between hosts and infections, is highlighted as one of the most advantageous strategies in the paper (Singh et al., 2022). Despite these benefits, the article also points out that employing an integrated omics approach has limitations as well. The complexity of the data generated, which necessitates sophisticated bioinformatics tools and methodologies, is one of the key problems. Another issue is that using various omics technologies could result in data that needs to be carefully validated to assure its accuracy (Singh et al., 2022).

Genome-wide screens are another omics technique used in this context to produce significant, high-quality data sets that can be utilised immediately to determine the genes that are involved in a certain process. The contributed article in this Research Topic has utilised genome-wide screening tool TraDIS (Transposon Directed Insertion-site Sequencing) to identify *Burkholderia pseudomallei* essential genes and predicted 492 genes carrying low insertion frequencies to be essential for bacteria survival. They emphasised that the conditional essential proteins of *B. pseudomallei* should give further information on the bacteria’s niche adaptability, pathogenesis, and virulence (Wong et al., 2022). Furthermore, another article has used whole genome sequencing to assess the virulence potential of Shiga toxin-producing *Escherichia coli* (STEC) O121 (Carter et al., 2022). The reduction in the number of genes associated with the Type III Secretion System is primarily responsible for the diversity in the virulence gene repertoire that was shown by the results. The findings showed diverse evolutionary lineages among the strains and revealed that some STEC strains had less pathogenicity potential.

In a related study, research paper on targeted proteomics was used to compare the quantitative virulomes of *Staphylococcus aureus* isolates from a large cohort of French patients with severe community-acquired staphylococcal pneumonia. The results indicated that the level of virulence components expressed *in vitro* by *S. aureus* may be correlated with the severity of the infection (Pivard et al., 2023).

Furthermore, another genomics study by Chen et al., 2023 explored the function of duodenal ulcer-promoting gene A (DupA) in gastropathy induced by *Helicobacter pylori*. They utilised 16S rRNA metagenome amplicon sequencing to analyse the microbiota DNA of the gastric mucosa and confirmed the expression of DupA from isolated *H. pylori* strains using PCR and qRT-PCR. According to their findings, high DupA expression in *H. pylori* is associated with a higher risk of erosive gastritis and a lesser degree of disruption to the gastric microbiota, demonstrating that DupA as a risk factor for erosive gastritis rather than gastric cancer (Chen et al., 2023). Therefore, measurements of functional activity for certain genes by RT-PCR are more powerful when integrated with traditional omics technologies that better capture genome and metabolome alterations, transcriptome perturbations, as well as proteome dynamics and post translational modifications (PTMs). Moreover, the combined knowledge from 16S rDNA and various omics approaches may enable better elucidation of the impact of the microbiota community on the corresponding hosts, paving the way to build support for new biological hypotheses.

In conclusion, the articles in this Research Topic have provided valuable perspectives using omics approaches that revolutionized the field of host-pathogen interactions. A thorough understanding of host-pathogen interactions is critical for the development of infectious disease therapies and prevention strategies.

## Author contributions

ARA prepared and wrote the editorial manuscript. KB and NR reviewed and edited the editorial manuscript. All authors contributed to the article and approved the submitted version.
